# P060 Alzheimer’s, vasculitis, or both? A diagnostic tightrope

**DOI:** 10.1093/rap/rkag113.090

**Published:** 2026-11-03

**Authors:** Elizabeth Emmett, Grant Perry, Iman Ali

**Affiliations:** Older Adult Psychiatry, Gloucestershire Health and Care NHS Foundation Trust, Cheltenham, United Kingdom; Department of Rheumatology, Gloucestershire Hospitals NHS Foundation Trust, Cheltenham, United Kingdom; Department of Rheumatology, Gloucestershire Hospitals NHS Foundation Trust, Cheltenham, United Kingdom

## Abstract

**Introduction:**

Giant cell arteritis (GCA) rarely presents with isolated vertebral artery involvement, and diagnosis is particularly challenging in patients with coexisting cognitive impairment. We present an atypical case of GCA in an 85-year-old woman with polymyalgia rheumatica (PMR) and steroid-induced psychiatric effects, where cognitive decline was likely driven by both Alzheimer’s disease and large vessel vasculitis. This case illustrates diagnostic complexity, challenges in steroid management in the elderly, and the importance of multidisciplinary decision-making.

**Case description:**

An 85-year-old Caucasian female was diagnosed with PMR in 2022, presenting with pelvic and shoulder girdle pain with raised inflammatory markers. Initial treatment with prednisolone improved her symptoms. She later developed generalised headaches and painful eye movements, raising suspicion of GCA. Despite a family history of GCA, GCA was considered unlikely due to the absence of classical features, normal temporal and axillary artery ultrasound, and lack of steroid response. Temporal artery biopsy was not considered based on clinical judgment and patient preference.

During corticosteroid treatment, she developed confusion, agitation, and anxiety, attributed to the steroids. Prednisolone was discontinued in 2023, and psychiatric management was initiated. By late 2023, cognitive decline persisted with a MOCA score of 24/30. Brain MRI and CT scans were inconclusive, leaving diagnostic uncertainty between AD, vasculitis-related cognitive impairment, and medication effects.

In February 2025, she experienced cognitive and functional decline requiring admission under the Mental Health Act (MHA). PET-CT brain imaging revealed increased uptake in the vertebral and external carotid arteries, suggesting large vessel vasculitis despite no active PMR or GCA symptoms. Whole-body PET-CT confirmed isolated vertebral artery involvement. Amyloid PET confirmed Alzheimer’s disease, supporting a dual diagnosis.

Following a multidisciplinary team discussion, which balanced the concerns about re-introducing steroids, her cognitive impairment and that large vessel vasculitis was likely contributing to her presentation, tocilizumab was initiated. There was an initial improvement in her headaches, although no change was seen otherwise. After 8 months of tocilizumab, transient headaches were observed during the day of each infusion prompting further reassessment, with a repeat ultrasound and follow-up PET-CT. These demonstrated no active vasculitis, suggesting disease control despite symptom recurrence. Tocilizumab was therefore discontinued and methotrexate was introduced as maintenance therapy.

**Discussion:**

Unlike a ‘typical’ case of GCA, characterised by temporal headaches, jaw claudication, abnormal markers, and confirmatory ultrasound or biopsy, this case is atypical with a normal ultrasound, no biopsy performed, and vertebral artery involvement. Her history of steroid-induced side-effects, alongside cognitive decline, prompted an MDT decision to initiate tocilizumab.

Concurrent Alzheimer’s disease likely caused the cognitive decline, but vasculitis may also have contributed, and this is a key insight for this case that should be considered by older adult psychiatrists and rheumatologists. It remains unclear how each pathology may have contributed to her presentation. The PET-CT played a vital role in identifying extracranial vascular involvement, whilst the amyloid PET confirmed dual pathophysiological processes highlighting the importance of using these investigations to guide diagnosis and management. This case required MDT decision making across psychiatry and rheumatology, which was unusual, but important for this patient’s care.

**Key learning points:**

• GCA may present atypically with vertebral artery involvement despite normal temporal imaging.

• Steroid-induced psychiatric effects may limit standard therapy; biologic alternatives should be considered.

• Cognitive decline should not be presumed to be solely neurodegenerative in origin.

• PET-CT is valuable for detecting atypical vasculitic involvement.

• Multidisciplinary collaboration is critical in complex cases.

